# Engagement with Care, Substance Use, and Adherence to Therapy in HIV/AIDS

**DOI:** 10.1155/2014/675739

**Published:** 2014-04-03

**Authors:** Patrice K. Nicholas, Suzanne Willard, Clinton Thompson, Carol Dawson-Rose, Inge B. Corless, Dean J. Wantland, Elizabeth F. Sefcik, Kathleen M. Nokes, Kenn M. Kirksey, Mary Jane Hamilton, William L. Holzemer, Carmen J. Portillo, Marta Rivero Mendez, Linda M. Robinson, Maria Rosa, Sarie P. Human, Yvette Cuca, Emily Huang, Mary Maryland, John Arudo, Lucille Sanzero Eller, Mark A. Stanton, MaryKate Driscoll, Joachim G. Voss, Shahnaz Moezzi

**Affiliations:** ^1^Global Health and Academic Partnerships, Division of Global Health Equity and Center for Nursing Excellence, Brigham and Women's Hospital, MGH Institute of Health Professions, 36 1st Avenue, Boston, MA 02129, USA; ^2^Advanced Practice Program, College of Nursing, Rutgers, The State University of New Jersey, Ackerson Hall, Room 362, 180 University Avenue, Newark, NJ 07102, USA; ^3^College of Public Health, The George Washington University, 2121 I Street NW, Washington, DC 20052, USA; ^4^Community Health Systems, University of California San Francisco School of Nursing, 2 Koret Way, N505, P.O. Box 0608, San Francisco, CA 94143-0608, USA; ^5^MGH Institute of Health Professions, 36 1st Avenue, Boston, MA 02129, USA; ^6^College of Nursing, Rutgers, The State University of New Jersey, Ackerson Hall, Room 330, 180 University Avenue, Newark, NJ 07102, USA; ^7^College of Nursing and Health Sciences, Texas A&M University-Corpus Christi, 6300 Ocean Drive, Unit 5805, Corpus Christi, TX 78411, USA; ^8^Hunter-Bellevue School of Nursing, Hunter College, City University of New York, 425 East 25 Street, P.O. Box 874, New York, NY 10010, USA; ^9^Nursing Strategic Initiatives, Harris Health System, Lyndon B. Johnson General Hospital, 5656 Kelley Street, Houston, TX 77026, USA; ^10^College of Nursing & Health Science, Texas A&M University-Corpus Christi, 6300 Ocean Drive, Corpus Christi, TX 78412, USA; ^11^School of Nursing, University of Puerto Rico Medical Sciences Campus, P.O. Box 365067, San Juan, PR 00936-5067, USA; ^12^San Diego State University School of Nursing, 5500 Campanile Drive, San Diego, CA 92182-4158, USA; ^13^Institute for Hispanic Health, National Council of La Raza, 1126 16th Street, Washington, DC 20036, USA; ^14^MPH Programme, Department of Health Studies, University of South Africa, Pretoria 0003, South Africa; ^15^Department of Health Systems Science, College of Nursing, University of Illinois at Chicago (UIC), 845 S. Damen Avenue, M/C 802, Chicago, IL 60302, USA; ^16^Rwanda Ministry of Health, Human Resources for Health Program, Division Head Nursing Services, Rwanda Military Hospital, Kigale, Rwanda; ^17^Clinical Nursing and Health Informatics, School of Nursing & Midwifery, Masinde Muliro University of Science and Technology, P.O. Box 190, Kakamega 50100, Kenya; ^18^College of Nursing, Rutgers, The State University of New Jersey, Ackerson Hall, Room 328, 180 University Avenue, Newark, NJ 07102, USA; ^19^Clemson University, Clemson, SC 29634, USA; ^20^Labs of Cognitive Neuroscience, Department of Developmental Medicine, Children's Hospital Medical Center and Brigham and Women's Hospital, 1 Autumn Street, 6th Floor, Boston, MA 02215, USA; ^21^Global Health Department, Behavioral Nursing & Health Systems, University of Washington, Health Sciences Building, Box 3572 66, Seattle, WA 98195, USA; ^22^University of Utah, 201 Presidents Circle, Salt Lake City, UT 84112, USA

## Abstract

Engagement with care for those living with HIV is aimed at establishing a strong relationship between patients and their health care provider and is often associated with greater adherence to therapy and treatment (Flickinger, Saha, Moore, and Beach, 2013). Substance use behaviors are linked with lower rates of engagement with care and medication adherence (Horvath, Carrico, Simoni, Boyer, Amico, and Petroli, 2013). This study is a secondary data analysis using a cross-sectional design from a larger randomized controlled trial (*n* = 775) that investigated the efficacy of a self-care symptom management manual for participants living with HIV. Participants were recruited from countries of Africa and the US. This study provides evidence that substance use is linked with lower self-reported engagement with care and adherence to therapy. Data on substance use and engagement are presented. Clinical implications of the study address the importance of utilizing health care system and policy factors to improve engagement with care.

## 1. Introduction

Engagement with care is a critical element of successful adaptation to chronic illness, particularly in HIV disease [1]. Establishing a successful patient-provider relationship may be linked with better outcomes for those living with HIV including lower viral loads, fewer comorbid illnesses, and slower progression to AIDS. Both alcohol and illicit drug use are common among HIV-infected patients [2] and may contribute to poorer adherence to antiretroviral therapy and less engagement with health care providers. Substance use, notably alcohol use, was found to predict HIV risk behavior and sensation seeking in a sample of men and women in South Africa [3] and contributes to lower utilization of and adherence to antiretroviral therapy [4]. It is known that medical providers have difficulty routinely addressing unhealthy behaviors and substance use, and these behaviors are not always addressed with patients in HIV treatment settings [5]. Recently, Horvath et al. [6] found that stimulant use was significantly correlated with missed HIV medical appointments and that fewer stimulant-using men in the study rated HIV medical care as a high priority.

### 1.1. Engagement with Care

Engagement with care is an important element of the patient-provider relationship and may be linked with health status outcomes, adherence to therapy, and chronic and comorbid illness in HIV disease [7]. A complex chronic illness such as HIV requires excellent care, communication, and a committed partnership of patient and provider [8, 9]. Dombrowski and colleagues [9] found that engagement with care was the only significant factor that was linked with both antiretroviral adherence and viral load suppression. Bankoff and colleagues [10] noted that positive patient-provider relationships were significantly correlated with better physical health and functioning and greater engagement in care. Rao et al. [11] noted that lack of engagement in HIV care presents a major clinical challenge in HIV care as well as a global public health challenge.

Mugavero et al. [12] examined health care system and policy factors that influence engagement in HIV clinical care. Their discussion included a socioecological framework that includes salient health care system and policy factors that influence engagement in HIV clinical care. They address the importance of communication and “synergy between funding and service agencies that provide HIV testing, prevention, treatment, and supportive services” (p. S238).

Since HIV disease is a chronic and complex illness that requires medication adherence, engagement with care, and symptom management across the HIV trajectory, the impact of, and the relationships among unhealthy substance use behaviors, relationships with care providers and the presence and severity of symptoms require exploration. Difficult symptoms are known to exist as a result of HIV disease and progression to AIDS, due to antiretroviral medication, and may also be linked to comorbid unhealthy behaviors. It is known that medical providers have difficulty routinely addressing unhealthy behaviors and substance use, in particular, is often not addressed with patients in HIV treatment settings [5].

### 1.2. Research Questions

The research questions were

* Is there a relationship between engagement with care, substance use behaviors, and adherence to therapy in HIV disease? Are there differences in adherence related to health care providers (nurse, advanced practice nurse/nurse practitioner, or physician) or by site? *

### 1.3. Purpose of the Study

As part of a larger randomized controlled study conducted by an international HIV/AIDS network on self-care for symptoms in HIV disease, the purpose of the present study was to examine the participants' perception of engagement with care, self-reported substance use, and adherence to therapy. The parent study was a randomized controlled trial of an HIV symptom management manual and its effectiveness in addressing symptoms in HIV disease. The prevalence of substance use behaviors including excessive alcohol use, illicit drug use, and cigarette smoking were examined as well as quality of life in HIV disease. Although the larger study was a randomized controlled trial of the use of a symptom management manual, the present study reports on the descriptive, cross-sectional data regarding engagement with care, adherence to therapy, and substance use behaviors. SPSS PC version 16.0 was used to analyze and categorize the study data. Quantitative methods were used to analyze and summarize symptom data related to unhealthy behaviors.

## 2. Materials and Methods

This is a secondary analysis of data from a randomized controlled trial to examine engagement with care, adherence, and unhealthy self-care behaviors in a sample of persons with HIV/AIDS. Data were collected in 14 sites in Africa (Nairobi, Kenya, and Pretoria, South Africa), Puerto Rico, and the United States (Boston, Chicago, Philadelphia, Salt Lake City, San Diego, San Francisco, and Texas). The settings included community-based organizations, university-based AIDS clinics, private practices, public and for-profit hospitals, residential and day care facilities, and home care services. Institutional review board (IRB) approval was obtained at each of the sites where data were collected as well as from the UCSF International Nursing Research Network IRB.

### 2.1. Participants

The sample for this study included HIV-infected individuals (*n* = 775) from the U.S. (Boston, 9.0%, Chicago 2.1%, Philadelphia, 13.8%, California 13.8%, Texas 24.5%, and Salt Lake City 9.0%), Pretoria, South Africa (6.2%), and Nairobi, Kenya (9.2%). Of the larger sample of 775 participants, a majority of the respondents reported substance use behaviors. Inclusion criteria for the study were that participants had to be (a) at least 18 years of age, (b) receiving HIV-related care at their respective facilities, (c) able to provide informed consent, and (d) able to complete the questionnaire independently or with the assistance of a research assistant.

### 2.2. Measures

#### 2.2.1. Demographic Survey

A 13-item self-report survey was utilized to collect demographic information and illness background data for the study. Questions addressed personal and environmental characteristics including age, gender, years of education, adequacy of income, and whether they had children among other variables. In addition, data on biological/physiological factors, such as whether participants had received an AIDS diagnosis or had any comorbidities, were also collected.

### 2.3. Engagement with Health Care Provider (HCP)

The health care provider engagement with care instrument (HCP) is a 13-item scale where clients rate the nature of their interactions with their health care providers on a four-point scale with 1 = always true and 4 = never true. A low score indicates greater provider engagement. The scale was submitted to a principal components factor analysis with varimax rotation. A one-factor solution emerged with an eigenvalue of 8.6 and explaining 66.5% of the variance. Cronbach's alpha reliability estimate was 0.96 [7].

### 2.4. Revised ACTG Reasons for Nonadherence to Medications (ACTGrev)

The ACTG revised is a self-report measure of reasons for missing medications [13] that was revised through a large randomized clinical control study on adherence. In this study, clients were asked to respond to reasons for missing medications using a 4-point Likert-type scale ranging from never to often, with a higher score denoting lesser adherence to their current medication regime. Factor analysis was performed on Chesney and colleagues' [13] original 14 item instrument, producing 2 factors with a total of nine items: pill taking problems (5 items) and forgetfulness (4 items). These nine items constitute the revised scale. Each factor or subscale can be summed separately and then collectively to create the total score. Cronbach's alphas for the 2 subscales and the total score ranged from 0.80 to 0.90.

### 2.5. Drug and Alcohol Use Scale (DAS)

This 35-item scale was derived from the Addiction Severity Index (ASI), which is the most widely used standardized instrument in assessing substance use [14]. The ASI is a semistructured interview containing 161 questions in seven categories that assess demographic information, medical status, employment and education, family and social background, psychiatric status, history of drug and alcohol use, and legal status. Test retest reliability coefficients of 0.83 over a 2-day period have been demonstrated with the whole tool. The 35-item drug and alcohol scale was transformed from a semistructured format to a self-report questionnaire for use in this study. This scale inquires about lifetime use patterns and drug use episodes specifically in the past 30 days. Administration time for the drug and alcohol scale has been estimated at about 5 minutes. Level of substance use will be determined by the number of times participants report using specific drugs in the past 30 days.

### 2.6. Data Analyses

Standard descriptive statistics were used to describe the demographic characteristics, illness background, engagement with care, adherence to therapy, and unhealthy behaviors for symptom management. The alpha level for all statistics was set at 0.05 and SPSS (version 18.0) was used for all data analyses. Means, standard deviations, and *t*-tests for levels of engagement with care and unhealthy behaviors were calculated. Significant differences between engagement with care, adherence, and unhealthy behaviors were examined.

## 3. Results

Demographic and illness background data are presented in Table 1. The sample was composed of 59% (*n* = 455) males, 38.5% (*n* = 296) females, and 2.2% (*n* = 17) who self-identified as transgender. Ages ranged from 20 to 72 years (*M* = 42.8, SD 9.6 years). The largest racial groups represented were African national and African American (43.6%; *n* = 335), Hispanic/Latino (27.8%; *n* = 214), and White/Anglo (21.2%; *n* = 163). Most of the participants had at least a high school education (70.9%; *n* = 544) although 78.2% (*n* = 604) indicated that their income was, at best, barely adequate. Forty-two percent (*n* = 322) of the participants had an AIDS diagnosis. The mean years of known HIV-positive diagnosis was 9.1 years and the majority of participants were currently taking antiretroviral medications (70.4%; *n* = 537) and had been taking antiretroviral medications for a mean number of 6.7 years. Their most recent CD4 counts ranged from 0 to 1200 with a mean CD4 count of 407; 62.7% (*n* = 470) reported that they had other comorbid medical conditions in addition to HIV/AIDS.

Several symptoms were significantly associated with unhealthy substance use behaviors. In particular, fatigue and emotional stress were correlated with all four substance use behaviors (heavy alcohol use, illicit drug use, marijuana, and tobacco). Of note is that nearly half of the sample (45.8%; *n* = 355) reported tobacco use and nearly 15% reported marijuana use (*n* = 111, 14.3%). Illicit drug use was self-reported by 2.6% of the sample (*n* = 98) and heavy alcohol use by 8.5% (*n* = 66).

**Figure 1:**
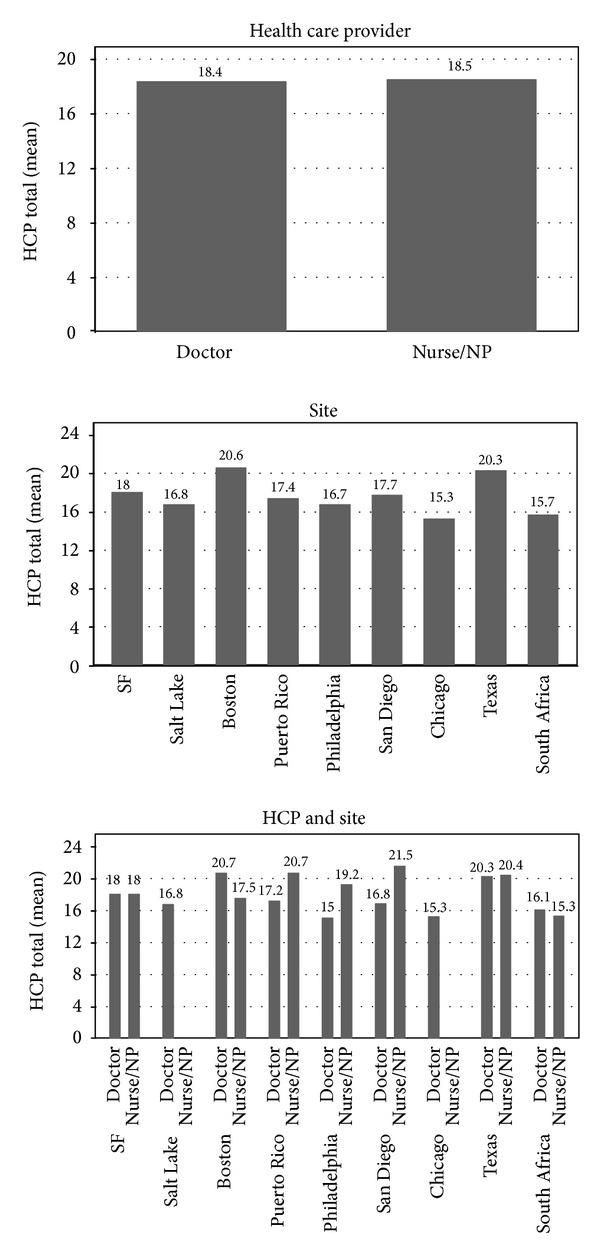
Baseline engagement with care (by health care Provider, site, and provider/site). Engagement with care is scaled such that lower scores denote better engagement.

Figure 1 displays the baseline engagement with care scores by health care provider, site, and provider/site. Respondents indicated that mean values on health care provider responses were not significantly different for those whose care was offered by physicians or those who had nurse providers (physician care *M* = 18.4; nurse care *M* = 18.5). There were also no significant differences in engagement with care by site. It is of note that the engagement with care data was not collected for this analysis in Kenya and thus analyses from countries of Africa are limited. In some sites, respondents indicated greater engagement with care when cared for by a physician including. Participants in Boston and South Africa had greater engagement with care reported by those who received care from nurse; however, none of the engagement with care scores were significantly different.

**Figure 2:**
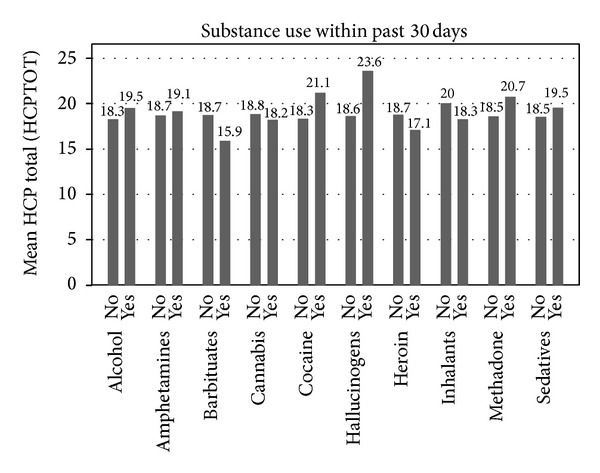
Baseline engagement with care vis-a-vis substance use within the past 30 days. Engagement with care is scaled such that lower scores denote better engagement.

In Figure 2, baseline engagement with care and substance use are displayed. The lowest scores of engagement with care were for those who used hallucinogens (*M* = 23.6) followed by cocaine users (*M* = 21.1). It is interesting to note that for those who used heroin, the mean engagement with care scores were low at 17.1. The lowest engagement with care score was for those using barbiturate medications. However none of the engagement with care scores were significantly different based on self-report of specific illicit drugs used by participants. The highest adherence scores and lowest reports of substance use were in South Africa. Not surprisingly, mean engagement with care scores were the highest in those who did not report any drug use. Regarding adherence to therapy, respondents in Puerto Rico had the poorest adherence to therapy. For those who had a nurse provider, the highest mean scores of adherence were in South Africa (0.111; *n* = 9), Philadelphia (5.35; *n* = 17), and Boston (6.5; *n* = 2). The lowest mean scores of adherence were Puerto Rico (11.1; *n* = 4), Texas (11.1; *n* = 14), and San Diego (8.25; *n* = 4).

It is of note that Wilcoxon *t*-tests indicated that symptom burden was significantly higher for those who reported use versus nonuse of alcohol (*P* < 0.001), tobacco (*P* < 0.001), illicit drugs (*P* < 0.0001), and marijuana (*P* < 0.004). Mean symptom burden for substance users for alcohol was 9.0, while nonusers reported a symptom burden of 6.6 (*P* < 0.001). For illicit drugs, mean symptom burden for users was 9.1, while nonusers reported a mean symptom burden score of 6.5 (*P* < 0.0001). Marijuana users reported a symptom burden of 8.3 and nonusers had a mean score of 6.5 (*P* < 0.004); tobacco users reported a mean symptom burden score of 7.6 and nonusers had a score of 6.1 (*P* < 0.001).

## 4. Discussion

In this study, substance use was associated with significantly lower scores of engagement with care and linked with poorer adherence to therapy. These results are important in addressing HIV care and providing support for patients across the trajectory of HIV disease. Although the sample was primarily male, the HIV epidemic increasingly affects female populations—particularly in the participants in South Africa. Symptoms that were most associated with unhealthy substance use behaviors were fatigue and emotional stress. Both were significantly correlated with all four substance use behaviors (heavy alcohol use, illicit drug use, marijuana, and tobacco). Tobacco use was self-reported by nearly half of the sample; thus education about the deleterious effects of these substances is critical for nurses and other health care providers. Many participants may also have become infected due to injection drug use, so assessment of continuing substance use by health care providers is critical. Drug addiction is universal; however, the factors leading to substance abuse are complex across cultures and communities. In our study, we found no significant differences in our analyses for US and non-US participants living with HIV on types of substance, demographics, education, or engagement with care variables.

Baseline engagement with care was not significantly different depending upon providers whether the provider was a nurse, advanced practice nurse, or physician providing care in most sites. It may be that HIV providers are a unique group in their commitment to their patients and HIV care. This may suggest that, even with complex patients including those who use substances, HIV providers have greater empathy, are attentive to the stigmatizing nature of the disease, and are committed to quality care and accessibility.

Symptom burden was significantly higher for those who reported use versus nonuse of alcohol, tobacco, illicit drugs, and marijuana. This finding is important to discuss with the patients living with HIV/AIDS. Many are likely using substances to alleviate symptoms and, yet, may have a greater number of symptoms as a result of substance use.

### 4.1. Recommendations for Future Research

These results offer important contributions to the care of those living with HIV and the importance of the relationship with the health care provider. It is essential that health care providers practicing in HIV settings incorporate assessment of substance use at each health care visit. Interventions specifically tailored for those with a history of substance use must also be utilized for this population. In particular, HIV disease may have occurred due to substance use and injection drugs. It is important that assessment of ongoing substance use be a key intervention at each visit with a provider. Evaluation of educational interventions by providers should be conducted at each HIV visit.

Future research should examine the HIV provider role in assessment of substance use in those living with HIV as well as substance use education. In addition, nurses and physicians should actively engage in the comprehensive plan of care as well as addressing the potential negative effects of substance use for those with HIV/AIDS. As individuals live longer with HIV disease as a chronic illness, other comorbid conditions frequently arise (diabetes and heart disease) and substance use may also add a complex dimension to their care. Further research is likely to uncover additional interventions that could support improved care of those with active and past substance users in leading healthy lifestyles with HIV disease.

## Figures and Tables

**Table 1:** Sample demographics.

Gender		
Male	455	59.2%
Female	296	38.5%
Transgender	17	2.2%
Age	42.8 years	SD = 9.6 (range 20–72)
Race		
Asian/Pacific Islander	11	1.4%
African national and American/black	335	43.6%
Hispanic/Latino	214	27.8%
Native American Indian	8	1.0%
White/Anglo (non-Hispanic)	163	21.2%
Other	37	4.8%
Highest education		
Grade school	223	29.1%
High school	318	41.5%
Technical/vocational school	143	18.6%
College	59	7.7%
Master's	20	2.6%
Doctorate	4	0.5%
# Children at home		
0	139	37.9%
1	81	22.1%
2	80	21.8%
3	31	8.4%
>4	36	9.8%
Data Collection Sites		
USA (10)		
California (3)	107	13.8%
Texas (3)	190	24.5%
Massachusetts (1)	69	9.0%
Utah (1)	69	9.0%
Illinois (1)	16	2.0%
Pennsylvania (1)	107	13.8%
Africa (2)		
South Africa (1)	48	6.2%
Kenya (1)	71	9.2%
Puerto Rico (2)		
San Juan (1)	70	9.0%
Vega Baja (1)	28	3.6%
AIDS diagnosis		
Yes	322	42.0%
No	408	53.2%
Don't know	37	4.8%
Taking HIV Meds now		
Yes	537	70.4%
No	226	29.6%
Other Medical Conditions		
Yes	470	62.7%
No	280	37.3%
Years HIV Positive	9.1 years	SD ± 6.6 (Range: 0–26)
Recent CD4 Count (if known)	407	SD ± 268 (Range: 0–1200)
Viral Load “Undetectable”	251	33.4%
Viral Load (Greater than 50 copies/mL) (*n* = 146)	50368	SD ± 135000 (Range: 50–750000)
Years on ARV Medications	6.7 years	SD ± 5.2 (Range: 0–20)
